# Crystal structure of tris­(2,2′-bi­pyridine)­cobalt(II) bis­(1,1,3,3-tetra­cyano-2-eth­oxy­propenide)

**DOI:** 10.1107/S2056989018018261

**Published:** 2019-01-04

**Authors:** Jamila Benabdallah, Zouaoui Setifi, Fatima Setifi, Habib Boughzala, Abderrahim Titi

**Affiliations:** a Laboratoire de Matériaux et Cristallochimie, Faculté des Sciences de Tunis, Université de Tunis El Manar, 2092 Manar II Tunis, Tunisia; b Département de Technologie, Faculté de Technologie, Université 20 Août 1955-Skikda, BP 26, Route d’El-Hadaiek, Skikda 21000, Algeria; c Laboratoire de Chimie, Ingénierie Moléculaire et Nanostructures (LCIMN), Université Ferhat Abbas Sétif 1, Sétif 19000, Algeria; d Laboratoire de Chimie Appliquée et Environnement (LCAE), Faculté des Sciences, Université Mohamed Premier, BP 524, 60000, Oujda, Morocco

**Keywords:** crystal structure, polynitrile ligand, ternary systems, hydrogen bonding, anion⋯π inter­actions

## Abstract

In the title compound, [Co(C_10_H_8_N_2_)_3_](C_9_H_5_N_4_O)_2_, the tris­(2,2′-bi­pyridine)­cobalt(II) dication lies across a twofold rotation axes in the space group *C*2/*c*. The N atoms of the three bi­pyridine ligands form a distorted octa­hedron around the cobalt ion. All the N atoms of the polynitrile 1,1,3,3-tetra­cyano-2-eth­oxy­propenide anions participate in C—H⋯N hydrogen bonds ensuring crystal cohesion and forming a three-dimensional structure. The structure is further stabilized by C—H⋯π(cation) and anion⋯π(cation) inter­actions.

## Abstract 2

Polynitrile anions are known for their ability to combine with transition metals and co-ligands to form ternary systems. Here we report on the crystal structure of tris­(2–2′-bi­pyridine)­cobalt(II) bis­(1,1,3,3-tetra­cyano-2-eth­oxy­propenide).

## Chemical context   

Ternary complexes of transition metals are mixed complexes where the transition-metal center is coordinated by more than one type of ligand (Gaamoune *et al.*, 2010 ▸; Setifi *et al.*, 2016 ▸; Yuste *et al.*, 2009 ▸). Organic polynitrile anions are among the compounds able to form this type of complex. In addition to their ability to create original structures and different coordination modes, these organic anions exhibit inter­esting behaviour thanks to their high electronic delocalization (Thétiot *et al.*, 2003 ▸; Setifi *et al.*, 2016 ▸) and magnetic properties (Benmansour *et al.*, 2008 ▸, 2010 ▸).

Several studies of polynitrile ternary complexes with different transition metals and different co-ligands have been realized (Benmansour *et al.*, 2008 ▸; Gaamoune *et al.*, 2010 ▸; Setifi *et al.*, 2013 ▸, 2014*b* ▸, 2017 ▸; Addala *et al.*, 2015 ▸). To synthesize such types of complexes we chose 2,2′-bi­pyridine as co-ligand and cobalt(II) as the transition metal, in view of its promising applications in therapy and imaging, as well as in dye-sensitized solar cells (Renfrew *et al.*, 2017 ▸; Yum *et al.*, 2012 ▸). The synthesis and structural study of the title compound (I) is reported here.
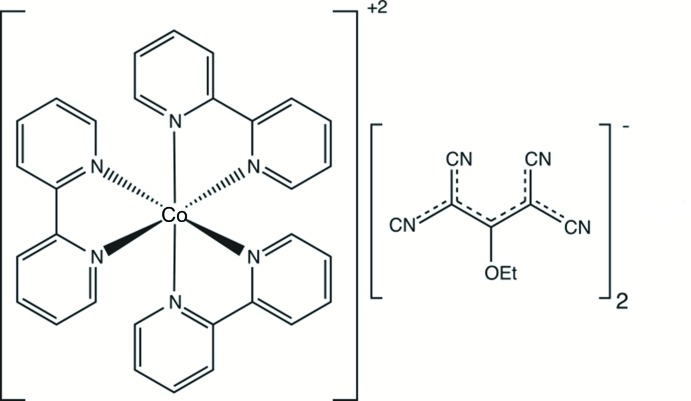


## Structural commentary   

**Figure 1:**
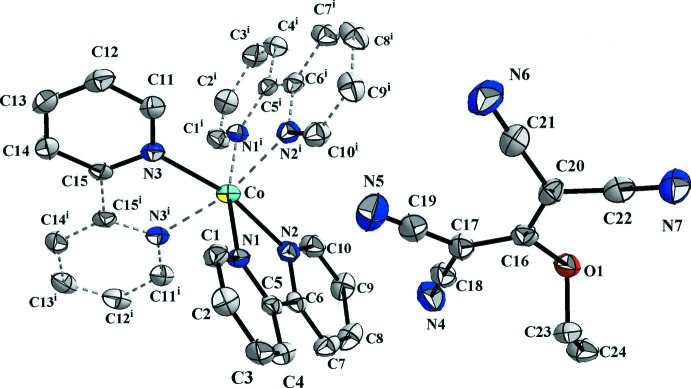
The independent components of compound (I), showing the atom-numbering scheme [symmetry code: (i) −*x* + 1, *y*, −*z* + 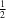]. Displacement ellipsoids are drawn at the 50% probability level. The hydrogen atoms have been omitted for clarity.

The asymmetric unit of the title compound (I) is illustrated in Fig. 1 ▸, and selected bond distances and angles are given in Table 1 ▸. The complex salt consists of half a tris­(2,2′-bi­pyridine)­cobalt(II) cation, the Co^II^ ion being located on a twofold rotation axis, and a 1,1,3,3-tetra­cyano-2-eth­oxy­propenide (tcnoet^−^), anion. The cobalt ion is ligated by the N atoms of the 2,2′-bi­pyridine ligands forming a slightly distorted octa­hedral coordination sphere; the Co1—N bond lengths vary from 2.122 (3) to 2.148 (3) Å. In the bpy (2,2′-bi­pyridine; N1/N2/C1–C10) unit, the pyridine rings are inclined to each other by 10.40 (16)°, while in the other bpy unit (involving atom N3) bis­ected by a twofold rotation axis the pyridine rings are coplanar. The observed distortion of the Co^II^coordination sphere is probably the consequence of the hydrogen bonding between the [Co(C_10_H_8_N_2_)_3_]^2+^ cation and the flexible tcnoet^−^ anion (see *Supra­molecular features*).

In the tcnoet^−^ anion, the six central C—C distances within the anion range from 1.382 (4) to 1.429 (5) Å while the C≡N distances vary from 1.138 (4) to 1.151 (4) Å (Table 1 ▸). As observed previously (Setifi *et al.*, 2016 ▸), these values confirm the electron delocalization in the tcnoet^−^ anion. The mean planes of the N≡C—C—C≡N moieties, N4/N5/C17–C19 and N6/N7/C20–C22, are inclined to each other by 31.7 (3)°.

## Supra­molecular features   

**Figure 2:**
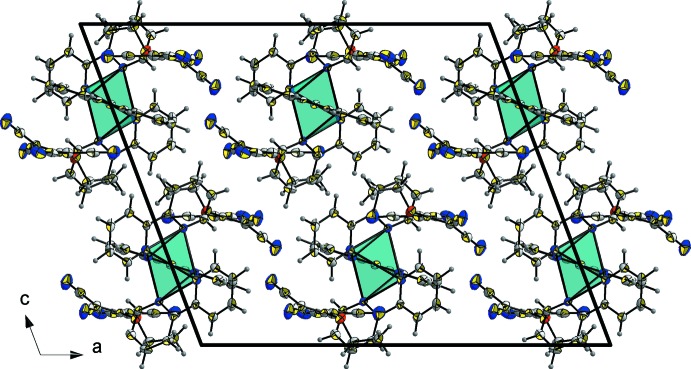
A view along the *b* axis of the crystal packing of compound (I).

The crystal packing of (I) is illustrated in Fig. 2 ▸. It can be described as an infinite three-dimensional association of the structural units linked by C—H⋯N hydrogen bonds and C—H⋯π and C≡N⋯π inter­actions; details of these inter­molecular inter­actions are given in Table 2 ▸.

**Figure 3:**
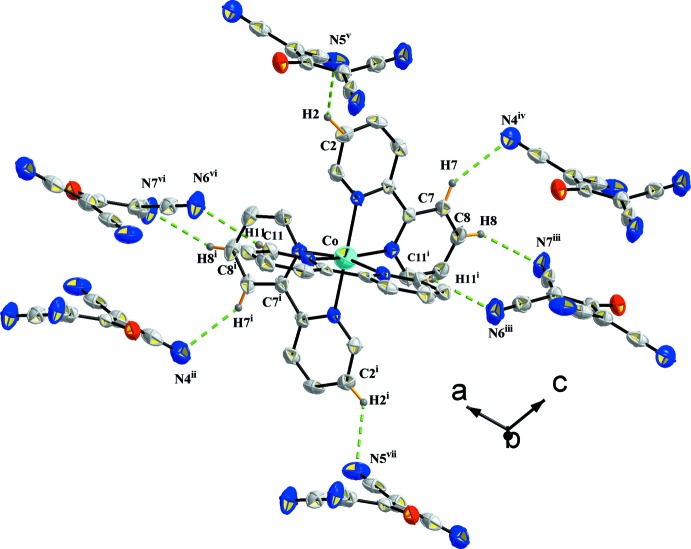
The hydrogen-bonding environment of the cation in the crystal of compound (I). Only H atoms involved in hydrogen bonding have been included. [Symmetry codes: (i) 1 − *x*, *y*, 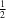 − *z*; (ii) *x*, 1 − *y*, *z* − 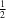; (iii) *x* − 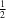, *y* + 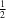, *z*; (iv) 1 − *x*, 1 − *y*, 1 − *z*; (v) 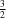 − *x*, 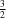 − *y*, 1 − *z*; (vi) 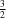 − *x*, 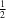 + *y*, 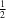 − *z*; (vii) −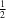 + *x*, 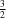 − *y*, −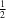 + *z*.]

**Figure 4:**
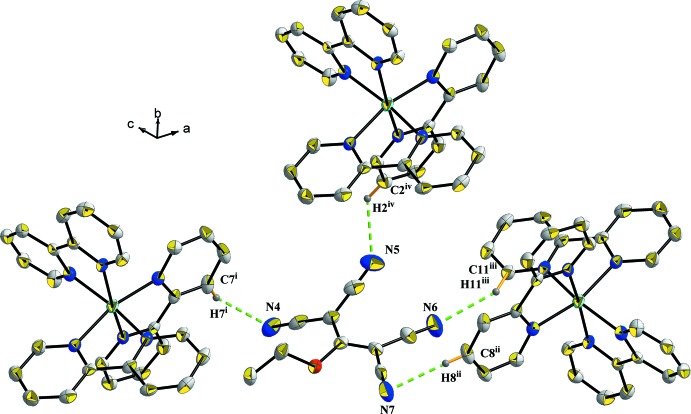
The hydrogen-bonding environment of the anion in compound (I). Only H atoms involved in hydrogen bonding have been included. [Symmetry codes: (i) 1 − *x*, 1 − *y*, 1 − *z*; (ii) 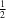 + *x*, −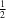 + *y*, *z*; (iii) 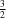 − *x*, −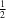 + *y*, 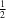 − *z*; (iv) 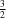 − *x*, 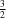 − *y*, 1 − *z*.]

The cations are surrounded by six tcnoet^−^ anions linked by eight C—H⋯N hydrogen bonds as shown in Fig. 3 ▸; the various symmetry codes are give in the figure caption. In the equatorial plane of the cobalt octa­hedron, two of the six tcnoet^−^ anions are doubly connected to the cationic units (N6, N7 and symmetry equivalents) via C8—H8⋯N7^iii^; C11^i^—H11^i^⋯N6^iii^ and their symmetric C8^i^—H8^i^⋯N7^iv^; C11—H11⋯N6^iv^. Four tcnoet^−^ anions are linked to atoms N4 and N5 (and symmetry equivalents) via C7—H7⋯N4^iv^, C2—H2⋯N5^v^, C7^i^—H7^i^⋯N4^ii^ and C2^i^—H2^i⋯^N5^vii^. One of the anions plays the role of a donor in the structural linkage. Indeed, one tcnoet^−^ anion is linked by an N⋯H—C inter­action to the same [Co(C_10_H_8_N_2_)_3_]^2+^ unit (via N6⋯H11^iii^—C11^iii^ and N7⋯C8^ii^—H8^ii^) and to two other cationic units by N4⋯H7^i^—C7^i^ and N5⋯H2^iv^—C2^iv^ inter­actions. This environment where the negative charge is delocalized over the central propenide unit as well as into the cyano groups is illustrated in Fig. 4 ▸. The various symmetry codes are give in the figure caption.

The crystal structure of (I) is reinforced by the presence of a C—H⋯π inter­action involving the methyl group of the propenide unit of the anion and the centroid of pyridine ring (N1/C1–C5) of the cation (C24—H24*B*⋯*Cg*1^vi^; see Table 2 ▸), and an anion⋯π inter­action between the centroid of pyridine ring (N2/C6–C10) of the cation and the nitro­gen atom N4 of the anion (C18—N4⋯*Cg*2; see Table 2 ▸).

## Database survey   

A search in the Cambridge Structural Database (CSD, version 5.39, last update August 2018; Groom *et al.*, 2016 ▸) using the query 1,1,3,3-tetra­cyano-2-eth­oxy­propenide gave 29 hits. 17 of these have the tcnoet^−^ anion associated with an organic cation to form a salt-like compound (Setifi *et al.*, 2015 ▸, 2014*a* ▸). The others have the anion associated to the metal ion acting as a coordinating ligand (Setifi *et al.*, 2009 ▸, 2013 ▸, 2017 ▸; Addala *et al.*, 2015 ▸; Gaamoune *et al.*, 2010 ▸). The closest structure to (I) found in this investigation is tris­(2,2′-bi­pyridine)­iron(II) bis­(1,1,3,3-tetra­cyano-2-eth­oxy­propenide) dihydrate (II) (CDS refcode CODZUS; Setifi *et al.*, 2014*b* ▸). The structural representation of (I) and (II) along the *b* axis points out some similarities in the cationic positions. However, in compound (II) the water mol­ecule links the tcnoet^−^ anion and the iron aggregate via O—H⋯N hydrogen bonds, forming chains, whereas in (I) the cation is directly linked to the anion via C—H⋯N hydrogen bonds forming a three-dimensional structure. There are no π–π stacking inter­actions in either compound, but in contrast to compound (I), compound (II) does not display any anion⋯π inter­actions. In the anion of (II), the mean planes of the N≡C—C—C≡N moieties are inclined to each other by *ca* 28.1° compared to 31.7 (3)° in (I).

## Synthesis and crystallization   

The title compound was synthesized solvothermally under autogenous pressure from a mixture of CoNO_3_·6H_2_O (29 mg, 0.1 mmol), 2,2-bi­pyridine (16 mg, 0.1 mmol) and K(tcnoet) (45 mg, 0.2 mmol) in water–ethanol (4:1 *v*/*v*, 20 cm^3^). This mixture was sealed in a Teflon-lined autoclave and held at 423 K for three days, and then cooled to ambient temperature at a rate of 10 K h^−1^ (yield: 54%). Colourless plate-like crystals of the title compound were selected directly from the synthesized product.

## Refinement   

Crystal data, data collection and structure refinement details are summarized in Table 3 ▸. All the hydrogen atoms could be located in difference-Fourier maps. During refinement they were included in calculated positions and treated as riding: C—H = 0.95–0.99 Å with *U*_iso_(H) = 1.5*U*_eq_(C-meth­yl) and 1.2*U*_eq_(C) for other H atoms.

## Supplementary Material

Crystal structure: contains datablock(s) I, Global. DOI: 10.1107/S2056989018018261/su5465sup1.cif

Structure factors: contains datablock(s) I. DOI: 10.1107/S2056989018018261/su5465Isup2.hkl

CCDC reference: 1887084

Additional supporting information:  crystallographic information; 3D view; checkCIF report

## Figures and Tables

**Table 1:** Selected geometric parameters (Å, °)

Co1—N1	2.122 (3)	C16—C17	1.393 (5)
Co1—N2	2.127 (3)	C16—C20	1.382 (4)
Co1—N3	2.148 (3)	C17—C19	1.415 (5)
N4—C18	1.151 (4)	C17—C18	1.417 (5)
N5—C19	1.151 (4)	C20—C22	1.423 (5)
N6—C21	1.138 (4)	C20—C21	1.429 (5)
N7—C22	1.145 (4)		
			
N1—Co1—N1^i^	167.82 (14)	N1—Co1—N3^i^	94.90 (9)
N1—Co1—N2	76.89 (10)	N2—Co1—N3^i^	93.21 (10)
N1—Co1—N2^i^	95.05 (10)	N3—Co1—N3^i^	76.48 (14)
N2—Co1—N2^i^	98.33 (13)	C19—C17—C18	117.4 (3)
N1—Co1—N3	94.66 (10)	C22—C20—C21	116.0 (3)
N2—Co1—N3	166.21 (9)		

Symmetry code: (i) 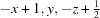.

**Table 2:** Hydrogen-bond geometry (Å, °) *Cg*1 and *Cg*2 are the centroids of N1/C1–C5 and N2/C6–C10 rings, respectively.

*D*—H⋯*A*	*D*—H	H⋯*A*	*D*⋯*A*	*D*—H⋯*A*
C7—H7⋯N4^ii^	0.95	2.52	3.406 (5)	155
C2—H2⋯N5^iii^	0.95	2.49	3.266 (5)	138
C11—H11⋯N6^iv^	0.95	2.61	3.302 (5)	130
C8—H8⋯N7^v^	0.95	2.48	3.268 (5)	140
C24—H24*B*⋯*Cg*1^vi^	0.98	2.84	3.757	155
C18—N4⋯*Cg*2	1.15 (1)	3.45 (1)	4.378 (4)	138 (1)

Symmetry codes: (ii) 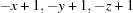; (iii) 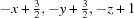; (iv) 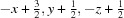; (v) 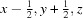; (vi) 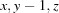.

**Table 3:** Experimental details

Crystal data	
Chemical formula	[Co(C_10_H_8_N_2_)_3_](C_9_H_5_N_4_O)_2_
*M*_r_	897.82
Crystal system, space group	Monoclinic, *C*2/*c*
Temperature (K)	162
*a*, *b*, *c* (Å)	22.335 (4), 10.9454 (17), 18.721 (3)
β (°)	110.691 (5)
*V* (Å^3^)	4281.4 (12)
*Z*	4
Radiation type	Mo *K*α
μ (mm^−1^)	0.46
Crystal size (mm)	0.21 × 0.18 × 0.06

Data collection	
Diffractometer	Bruker APEXII CCD
Absorption correction	Multi-scan (*SADABS*; Bruker, 2009 ▸)
*T*_min_, *T*_max_	0.643, 0.755
No. of measured, independent and observed [*I* > 2σ(*I*)] reflections	16461, 3929, 2190
*R*_int_	0.084
(sin θ/λ)_max_ (Å^−1^)	0.606

Refinement	
*R*[*F* ^2^ > 2σ(*F* ^2^)], *wR*(*F* ^2^), *S*	0.055, 0.109, 0.96
No. of reflections	3929
No. of parameters	295
H-atom treatment	H-atom parameters constrained
Δρ_max_, Δρ_min_ (e Å^−3^)	0.47, −0.58

Computer programs: *APEX2* and *SAINT* (Bruker, 2009 ▸), *SHELXS97* (Sheldrick, 2008 ▸), *DIAMOND* (Brandenburg, 2006 ▸), *SHELXL2018* (Sheldrick, 2015 ▸), *PLATON* (Spek, 2009 ▸) and *publCIF* (Westrip, 2010 ▸).

## supplementary crystallographic information

### Crystal data

**Table:**

[Co(C_10_H_8_N_2_)_3_](C_9_H_5_N_4_O)_2_	*F*(000) = 1852
*M**_r_* = 897.82	*D*_x_ = 1.393 Mg m^−^^3^
Monoclinic, *C*2/*c*	Mo *K*α radiation, λ = 0.71073 Å
*a* = 22.335 (4) Å	Cell parameters from 289 reflections
*b* = 10.9454 (17) Å	θ = 2.0–26.5°
*c* = 18.721 (3) Å	µ = 0.46 mm^−^^1^
β = 110.691 (5)°	*T* = 162 K
*V* = 4281.4 (12) Å^3^	Plate, colourless
*Z* = 4	0.21 × 0.18 × 0.06 mm

### Data collection

**Table:**

Bruker APEXII CCD diffractometer	3929 independent reflections
Radiation source: fine-focus sealed tube	2190 reflections with *I* > 2σ(*I*)
Graphite monochromator	*R*_int_ = 0.084
φ and ω scans	θ_max_ = 25.5°, θ_min_ = 2.0°
Absorption correction: multi-scan (SADABS; Bruker, 2009)	*h* = −18→26
*T*_min_ = 0.643, *T*_max_ = 0.755	*k* = −12→13
16461 measured reflections	*l* = −22→22

### Refinement

**Table:**

Refinement on *F*^2^	Primary atom site location: structure-invariant direct methods
Least-squares matrix: full	Secondary atom site location: difference Fourier map
*R*[*F*^2^ > 2σ(*F*^2^)] = 0.055	Hydrogen site location: inferred from neighbouring sites
*wR*(*F*^2^) = 0.109	H-atom parameters constrained
*S* = 0.96	*w* = 1/[σ^2^(*F*_o_^2^) + (0.0452*P*)^2^] where *P* = (*F*_o_^2^ + 2*F*_c_^2^)/3
3929 reflections	(Δ/σ)_max_ = 0.001
295 parameters	Δρ_max_ = 0.47 e Å^−^^3^
0 restraints	Δρ_min_ = −0.58 e Å^−^^3^

### Special details

**Table:**

Geometry. All esds (except the esd in the dihedral angle between two l.s. planes) are estimated using the full covariance matrix. The cell esds are taken into account individually in the estimation of esds in distances, angles and torsion angles; correlations between esds in cell parameters are only used when they are defined by crystal symmetry. An approximate (isotropic) treatment of cell esds is used for estimating esds involving l.s. planes.

### Fractional atomic coordinates and isotropic or equivalent isotropic displacement parameters (Å^2^)

**Table:**

	*x*	*y*	*z*	*U*_iso_*/*U*_eq_	
Co1	0.500000	0.81636 (6)	0.250000	0.0263 (2)	
N1	0.56363 (12)	0.7958 (2)	0.36434 (14)	0.0253 (7)	
N2	0.45167 (13)	0.6893 (2)	0.29657 (15)	0.0279 (7)	
N3	0.54955 (12)	0.9705 (2)	0.22633 (14)	0.0268 (7)	
C1	0.62261 (16)	0.8398 (3)	0.39281 (19)	0.0308 (9)	
H1	0.637978	0.886810	0.360240	0.037*	
C2	0.66287 (17)	0.8212 (3)	0.46700 (19)	0.0337 (9)	
H2	0.705215	0.852778	0.485105	0.040*	
C3	0.63939 (18)	0.7549 (3)	0.51399 (19)	0.0367 (9)	
H3	0.665527	0.740997	0.565732	0.044*	
C4	0.57842 (17)	0.7090 (3)	0.4862 (2)	0.0351 (9)	
H4	0.561858	0.664173	0.518486	0.042*	
C5	0.54094 (16)	0.7289 (3)	0.40983 (19)	0.0249 (8)	
C6	0.47718 (16)	0.6754 (3)	0.37336 (18)	0.0252 (8)	
C7	0.44605 (17)	0.6097 (3)	0.4125 (2)	0.0343 (9)	
H7	0.464431	0.601808	0.466467	0.041*	
C8	0.38825 (18)	0.5556 (3)	0.3731 (2)	0.0400 (10)	
H8	0.366063	0.511209	0.399616	0.048*	
C9	0.36287 (17)	0.5663 (3)	0.2952 (2)	0.0373 (10)	
H9	0.323671	0.527270	0.266767	0.045*	
C10	0.39518 (17)	0.6343 (3)	0.2592 (2)	0.0357 (9)	
H10	0.376978	0.643127	0.205269	0.043*	
C11	0.59964 (16)	0.9660 (3)	0.20301 (18)	0.0339 (9)	
H11	0.615396	0.888083	0.195848	0.041*	
C12	0.62955 (17)	1.0681 (3)	0.18886 (18)	0.0365 (10)	
H12	0.665344	1.060954	0.172932	0.044*	
C13	0.60629 (17)	1.1811 (3)	0.19837 (18)	0.0389 (9)	
H13	0.625453	1.253361	0.188249	0.047*	
C14	0.55520 (16)	1.1883 (3)	0.22259 (17)	0.0324 (9)	
H14	0.539026	1.265594	0.230061	0.039*	
C15	0.52721 (15)	1.0812 (3)	0.23614 (16)	0.0258 (8)	
N4	0.53638 (17)	0.3989 (3)	0.39931 (18)	0.0526 (10)	
N5	0.72153 (17)	0.5387 (3)	0.40013 (18)	0.0560 (10)	
N6	0.77126 (16)	0.3265 (3)	0.3033 (2)	0.0611 (10)	
N7	0.75554 (15)	−0.0297 (3)	0.39493 (19)	0.0508 (10)	
C16	0.66932 (16)	0.2352 (3)	0.39977 (18)	0.0297 (9)	
C17	0.65001 (18)	0.3558 (3)	0.40154 (19)	0.0341 (9)	
C18	0.5876 (2)	0.3814 (3)	0.4007 (2)	0.0396 (10)	
C19	0.6894 (2)	0.4565 (4)	0.4002 (2)	0.0404 (10)	
C20	0.71916 (16)	0.1944 (3)	0.37852 (18)	0.0308 (9)	
C21	0.74828 (17)	0.2690 (3)	0.3373 (2)	0.0385 (10)	
C22	0.73881 (17)	0.0699 (4)	0.3883 (2)	0.0340 (10)	
O1	0.63526 (11)	0.14388 (19)	0.41668 (12)	0.0346 (6)	
C23	0.62658 (17)	0.1448 (3)	0.49019 (18)	0.0382 (10)	
H23A	0.630478	0.229294	0.510234	0.046*	
H23B	0.659880	0.094019	0.527312	0.046*	
C24	0.56139 (16)	0.0949 (3)	0.4795 (2)	0.0425 (10)	
H24C	0.555584	0.091760	0.529008	0.064*	
H24B	0.557384	0.012409	0.457996	0.064*	
H24A	0.528652	0.147927	0.444679	0.064*	

### Atomic displacement parameters (Å^2^)

**Table:**

	*U*^11^	*U*^22^	*U*^33^	*U*^12^	*U*^13^	*U*^23^
Co1	0.0292 (4)	0.0314 (4)	0.0204 (4)	0.000	0.0115 (3)	0.000
N1	0.0276 (18)	0.0295 (18)	0.0211 (16)	−0.0019 (14)	0.0115 (14)	0.0013 (13)
N2	0.0270 (18)	0.0300 (17)	0.0288 (17)	−0.0003 (15)	0.0122 (15)	−0.0019 (15)
N3	0.0298 (18)	0.0328 (18)	0.0195 (16)	−0.0012 (14)	0.0106 (14)	−0.0005 (13)
C1	0.031 (2)	0.033 (2)	0.029 (2)	−0.0045 (18)	0.0109 (19)	0.0011 (17)
C2	0.028 (2)	0.036 (2)	0.033 (2)	0.0006 (19)	0.0062 (19)	−0.002 (2)
C3	0.040 (3)	0.041 (2)	0.020 (2)	0.001 (2)	0.0000 (19)	−0.0035 (18)
C4	0.041 (3)	0.039 (2)	0.026 (2)	0.000 (2)	0.014 (2)	0.0006 (18)
C5	0.028 (2)	0.025 (2)	0.022 (2)	0.0053 (16)	0.0107 (18)	−0.0023 (16)
C6	0.026 (2)	0.0260 (19)	0.027 (2)	0.0087 (17)	0.0124 (17)	0.0013 (17)
C7	0.032 (2)	0.041 (2)	0.036 (2)	0.0085 (19)	0.020 (2)	0.0092 (19)
C8	0.032 (3)	0.043 (3)	0.053 (3)	0.007 (2)	0.024 (2)	0.016 (2)
C9	0.029 (2)	0.033 (2)	0.053 (3)	−0.0024 (18)	0.019 (2)	0.003 (2)
C10	0.032 (2)	0.040 (2)	0.036 (2)	−0.0057 (19)	0.013 (2)	−0.0067 (18)
C11	0.040 (2)	0.036 (2)	0.029 (2)	0.0014 (19)	0.016 (2)	0.0026 (17)
C12	0.034 (2)	0.051 (3)	0.025 (2)	−0.011 (2)	0.0106 (19)	0.0020 (19)
C13	0.047 (3)	0.040 (3)	0.029 (2)	−0.013 (2)	0.013 (2)	0.002 (2)
C14	0.040 (2)	0.031 (2)	0.0213 (19)	0.001 (2)	0.0045 (18)	0.0001 (17)
C15	0.031 (2)	0.031 (2)	0.0107 (18)	−0.0023 (17)	0.0021 (16)	−0.0018 (15)
N4	0.059 (3)	0.048 (2)	0.052 (2)	0.014 (2)	0.022 (2)	−0.0022 (17)
N5	0.060 (3)	0.046 (2)	0.044 (2)	−0.009 (2)	−0.003 (2)	0.0049 (19)
N6	0.061 (3)	0.059 (2)	0.077 (3)	−0.005 (2)	0.041 (2)	0.015 (2)
N7	0.045 (2)	0.044 (2)	0.076 (3)	0.0008 (19)	0.037 (2)	0.011 (2)
C16	0.033 (2)	0.035 (2)	0.020 (2)	−0.0044 (18)	0.0068 (18)	−0.0033 (17)
C17	0.040 (3)	0.030 (2)	0.029 (2)	0.0031 (19)	0.009 (2)	−0.0003 (17)
C18	0.054 (3)	0.035 (2)	0.026 (2)	0.008 (2)	0.008 (2)	−0.0022 (18)
C19	0.048 (3)	0.038 (3)	0.024 (2)	0.007 (2)	−0.001 (2)	0.002 (2)
C20	0.029 (2)	0.031 (2)	0.031 (2)	−0.0065 (19)	0.0084 (18)	0.0022 (18)
C21	0.032 (2)	0.040 (2)	0.041 (3)	−0.0022 (19)	0.010 (2)	0.005 (2)
C22	0.023 (2)	0.043 (3)	0.039 (2)	−0.006 (2)	0.0158 (19)	0.002 (2)
O1	0.0428 (16)	0.0332 (15)	0.0343 (15)	−0.0057 (12)	0.0218 (13)	−0.0053 (11)
C23	0.049 (3)	0.044 (2)	0.025 (2)	0.0068 (19)	0.018 (2)	0.0047 (17)
C24	0.043 (3)	0.054 (3)	0.038 (2)	0.001 (2)	0.023 (2)	0.003 (2)

### Geometric parameters (Å, º)

**Table:**

Co1—N1	2.122 (3)	C10—H10	0.9500
Co1—N1^i^	2.122 (3)	C11—C12	1.375 (4)
Co1—N2	2.127 (3)	C11—H11	0.9500
Co1—N2^i^	2.127 (3)	C12—C13	1.377 (5)
Co1—N3	2.148 (3)	C12—H12	0.9500
Co1—N3^i^	2.148 (3)	C13—C14	1.371 (4)
N1—C1	1.325 (4)	C13—H13	0.9500
N1—C5	1.350 (4)	C14—C15	1.393 (4)
N2—C10	1.349 (4)	C14—H14	0.9500
N2—C6	1.355 (4)	C15—C15^i^	1.481 (6)
N3—C11	1.338 (4)	N4—C18	1.151 (4)
N3—C15	1.347 (4)	N5—C19	1.151 (4)
C1—C2	1.377 (4)	N6—C21	1.138 (4)
C1—H1	0.9500	N7—C22	1.145 (4)
C2—C3	1.379 (4)	C16—O1	1.359 (4)
C2—H2	0.9500	C16—C17	1.393 (5)
C3—C4	1.370 (4)	C16—C20	1.382 (4)
C3—H3	0.9500	C17—C19	1.415 (5)
C4—C5	1.394 (4)	C17—C18	1.417 (5)
C4—H4	0.9500	C20—C22	1.423 (5)
C5—C6	1.466 (4)	C20—C21	1.429 (5)
C6—C7	1.378 (4)	O1—C23	1.456 (3)
C7—C8	1.374 (5)	C23—C24	1.501 (4)
C7—H7	0.9500	C23—H23A	0.9900
C8—C9	1.370 (5)	C23—H23B	0.9900
C8—H8	0.9500	C24—H24C	0.9800
C9—C10	1.369 (4)	C24—H24B	0.9800
C9—H9	0.9500	C24—H24A	0.9800
			
N1—Co1—N1^i^	167.82 (14)	C10—C9—C8	118.6 (3)
N1—Co1—N2	76.89 (10)	C10—C9—H9	120.7
N1^i^—Co1—N2	95.05 (10)	C8—C9—H9	120.7
N1—Co1—N2^i^	95.05 (10)	N2—C10—C9	123.1 (3)
N1^i^—Co1—N2^i^	76.89 (10)	N2—C10—H10	118.5
N2—Co1—N2^i^	98.33 (13)	C9—C10—H10	118.5
N1—Co1—N3	94.66 (10)	N3—C11—C12	123.5 (3)
N1^i^—Co1—N3	94.90 (9)	N3—C11—H11	118.2
N2—Co1—N3	166.21 (9)	C12—C11—H11	118.2
N2^i^—Co1—N3	93.21 (10)	C11—C12—C13	118.3 (3)
N1—Co1—N3^i^	94.90 (9)	C11—C12—H12	120.9
N1^i^—Co1—N3^i^	94.66 (9)	C13—C12—H12	120.9
N2—Co1—N3^i^	93.21 (10)	C14—C13—C12	119.4 (3)
N2^i^—Co1—N3^i^	166.21 (9)	C14—C13—H13	120.3
N3—Co1—N3^i^	76.48 (14)	C12—C13—H13	120.3
C1—N1—C5	119.0 (3)	C13—C14—C15	119.5 (3)
C1—N1—Co1	125.7 (2)	C13—C14—H14	120.3
C5—N1—Co1	115.3 (2)	C15—C14—H14	120.3
C10—N2—C6	117.8 (3)	N3—C15—C14	121.3 (3)
C10—N2—Co1	126.6 (2)	N3—C15—C15^i^	115.92 (17)
C6—N2—Co1	115.1 (2)	C14—C15—C15^i^	122.74 (19)
C11—N3—C15	118.1 (3)	O1—C16—C20	113.8 (3)
C11—N3—Co1	126.1 (2)	O1—C16—C17	119.2 (3)
C15—N3—Co1	115.8 (2)	C20—C16—C17	126.9 (3)
N1—C1—C2	123.6 (3)	C16—C17—C19	122.6 (3)
N1—C1—H1	118.2	C16—C17—C18	119.9 (3)
C2—C1—H1	118.2	C19—C17—C18	117.4 (3)
C1—C2—C3	117.5 (3)	N4—C18—C17	178.0 (4)
C1—C2—H2	121.3	N5—C19—C17	179.1 (4)
C3—C2—H2	121.3	C16—C20—C22	121.0 (3)
C4—C3—C2	120.1 (3)	C16—C20—C21	122.5 (3)
C4—C3—H3	120.0	C22—C20—C21	116.0 (3)
C2—C3—H3	120.0	N6—C21—C20	178.6 (4)
C3—C4—C5	119.3 (3)	N7—C22—C20	178.1 (4)
C3—C4—H4	120.4	C16—O1—C23	119.2 (2)
C5—C4—H4	120.4	O1—C23—C24	108.6 (3)
N1—C5—C4	120.5 (3)	O1—C23—H23A	110.0
N1—C5—C6	116.2 (3)	C24—C23—H23A	110.0
C4—C5—C6	123.2 (3)	O1—C23—H23B	110.0
N2—C6—C7	121.4 (3)	C24—C23—H23B	110.0
N2—C6—C5	115.1 (3)	H23A—C23—H23B	108.4
C7—C6—C5	123.4 (3)	C23—C24—H24C	109.5
C8—C7—C6	119.6 (3)	C23—C24—H24B	109.5
C8—C7—H7	120.2	H24C—C24—H24B	109.5
C6—C7—H7	120.2	C23—C24—H24A	109.5
C9—C8—C7	119.5 (3)	H24C—C24—H24A	109.5
C9—C8—H8	120.3	H24B—C24—H24A	109.5
C7—C8—H8	120.3		
			
C5—N1—C1—C2	0.1 (5)	Co1—N2—C10—C9	−170.9 (2)
Co1—N1—C1—C2	−178.1 (2)	C8—C9—C10—N2	1.5 (5)
N1—C1—C2—C3	−1.3 (5)	C15—N3—C11—C12	0.2 (5)
C1—C2—C3—C4	0.8 (5)	Co1—N3—C11—C12	−179.9 (2)
C2—C3—C4—C5	0.8 (5)	N3—C11—C12—C13	−0.7 (5)
C1—N1—C5—C4	1.6 (4)	C11—C12—C13—C14	1.0 (5)
Co1—N1—C5—C4	180.0 (2)	C12—C13—C14—C15	−0.9 (5)
C1—N1—C5—C6	−175.6 (3)	C11—N3—C15—C14	0.0 (5)
Co1—N1—C5—C6	2.7 (3)	Co1—N3—C15—C14	−179.9 (2)
C3—C4—C5—N1	−2.1 (5)	C11—N3—C15—C15^i^	−178.5 (3)
C3—C4—C5—C6	175.0 (3)	Co1—N3—C15—C15^i^	1.6 (4)
C10—N2—C6—C7	−1.4 (5)	C13—C14—C15—N3	0.4 (5)
Co1—N2—C6—C7	170.8 (2)	C13—C14—C15—C15^i^	178.7 (3)
C10—N2—C6—C5	175.2 (3)	O1—C16—C17—C19	166.7 (3)
Co1—N2—C6—C5	−12.6 (3)	C20—C16—C17—C19	−16.8 (6)
N1—C5—C6—N2	6.6 (4)	O1—C16—C17—C18	−16.6 (5)
C4—C5—C6—N2	−170.6 (3)	C20—C16—C17—C18	159.9 (3)
N1—C5—C6—C7	−176.8 (3)	O1—C16—C20—C22	−11.2 (5)
C4—C5—C6—C7	6.0 (5)	C17—C16—C20—C22	172.1 (3)
N2—C6—C7—C8	0.8 (5)	O1—C16—C20—C21	160.7 (3)
C5—C6—C7—C8	−175.6 (3)	C17—C16—C20—C21	−16.0 (6)
C6—C7—C8—C9	1.0 (5)	C20—C16—O1—C23	125.8 (3)
C7—C8—C9—C10	−2.1 (5)	C17—C16—O1—C23	−57.2 (4)
C6—N2—C10—C9	0.3 (5)	C16—O1—C23—C24	145.0 (3)

Symmetry code: (i) −*x*+1, *y*, −*z*+1/2.

### Hydrogen-bond geometry (Å, º)

*Cg*1 and *Cg*2 are the centroids of N1/C1–C5 and N2/C6–C10 rings, respectively.

**Table:**

*D*—H···*A*	*D*—H	H···*A*	*D*···*A*	*D*—H···*A*
C7—H7···N4^ii^	0.95	2.52	3.406 (5)	155
C2—H2···N5^iii^	0.95	2.49	3.266 (5)	138
C11—H11···N6^iv^	0.95	2.61	3.302 (5)	130
C8—H8···N7^v^	0.95	2.48	3.268 (5)	140
C24—H24*B*···*Cg*1^vi^	0.98	2.84	3.757	155
C18—N4···*Cg*2	1.15 (1)	3.45 (1)	4.378 (4)	138 (1)

Symmetry codes: (ii) −*x*+1, −*y*+1, −*z*+1; (iii) −*x*+3/2, −*y*+3/2, −*z*+1; (iv) −*x*+3/2, *y*+1/2, −*z*+1/2; (v) *x*−1/2, *y*+1/2, *z*; (vi) *x*, *y*−1, *z*.
